# Application of Pipeline Embolization Device for Iatrogenic Pseudoaneurysms of the Extracranial Vertebral Artery: A Case Report and Systematic Review of the Literature

**DOI:** 10.7759/cureus.356

**Published:** 2015-10-19

**Authors:** Parviz Dolati, Daniel G Eichberg, Ajith Thomas, Christopher S Ogilvy

**Affiliations:** 1 Neurosurgery, Boston University School of Medicine; 2 Neurosurgery, BIDMC Harvard Medical School

**Keywords:** vertebral artery, pipeline embolization device, iatrogenic, pseudoaneurysm, flow diverting stent

## Abstract

Traumatic pseudoaneurysms of the vertebral artery (VA) are uncommon vascular lesions and definitive management is often challenging. Between 0% and 8% of craniocervical fusions are complicated by VA injury. In these cases, preserving the vertebral artery while treating the pseudoaneurysm is the goal of any treatment option. We describe the second known case of a patient with and iatrogenic extracranial vertebral artery pseudoaneurysm treated effectively using the Pipeline Embolization Device (PED) (Ev3 Neurovascular, Irvine, CA). Although there have been only two cases reported, the use of flow-diverting stents appears to be efficacious for the treatment of non-actively bleeding traumatic pseudoaneurysms.

## Introduction

Vertebral artery (VA) pseudoaneurysms arise most commonly due to blunt or penetrating trauma, following surgery, collagen vascular disease, or arterial dissection [1]. Because it emerges from the transverse foramen and courses adjacent to C1, the extracranial segment (V3) of the VA is the most susceptible to iatrogenic damage. Between 0% and 8% of craniocervical fusion surgeries are complicated by VA injury [2-7], which may result in arterial ischemic injury, severe hemorrhage, and death. Rarely, iatrogenic VA injury has been reported to lead to pseudoaneurysm development. Despite the potential for such pseudoaneurysms to resolve spontaneously, they have been shown to rupture in 31% to 54% of cases [8]. Thus, it is critical to rapidly diagnose and treat these vascular lesions to minimize the risk of potential grave morbidity and mortality.

Iatrogenic pseudoaneurysms are most commonly treated by surgical or endovascular options, either with or without revascularization [8-9]. Additionally, the use of stent grafts, covered stents, coil embolization, and onyx embolization has been reported [10-13]. Flow-diverting stents, such as the Pipeline Embolization Device (PED) (Ev3 Neurovascular, Irvine, CA), have been gaining increasing interest in the treatment of traumatic pseudoaneurysms [14]. We describe the second reported case of iatrogenic vertebral artery pseudoaneurysm that was treated effectively using the PED. We successfully reconstructed the injured VA, isolated the pseudoaneurysm from circulation, and maintained blood flow through the parent artery.

## Materials and methods

### Literature analysis

A comprehensive review of the literature was performed using the keywords “Pipeline Embolization Device,” “vertebral artery traumatic pseudoaneurysm,” “flow-diverting stent,” and “iatrogenic extracranial pseudoaneurysm” alone or together to search PubMed, Ovid Medline, Ovid EMBASE, Scopus, Web of Science database, and all neurosurgical journals. Inclusion criteria were the following: English language and presentation of patients with iatrogenic pseudoaneurysms of the vertebral artery treated with the Pipeline Embolization Device. There were no exclusion criteria.

## Results

### Case report                

**Figure 1:**
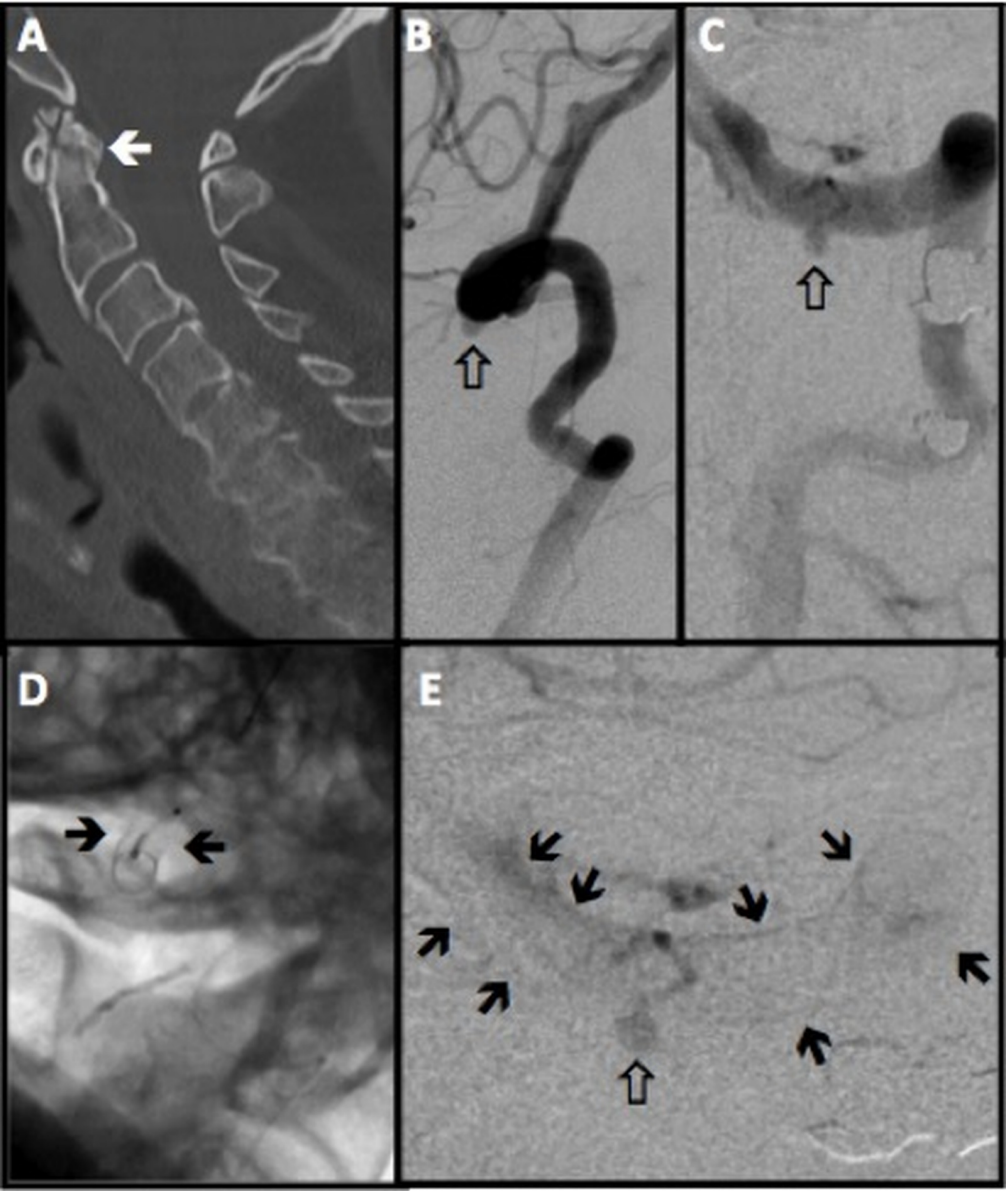
Vertebral Artery Imaging Before and After Pipeline Deployment Panel A, sagittal computed tomography angiography showing a non-union C2 odontoid fracture. Panels B and C, the lateral and anterior-posterior (AP) view of the left vertebral artery angiography, respectively. The white arrows are pointing to the pseudoaneurysm at the left V3 segment. Panels D and E show a deployed Pipeline in lateral and AP projections across the pseudoaneurysm, respectively (black arrows). The white arrow in panel E points to the stagnation of the blood flow inside the pseudoaneurysm.

**Figure 2:**
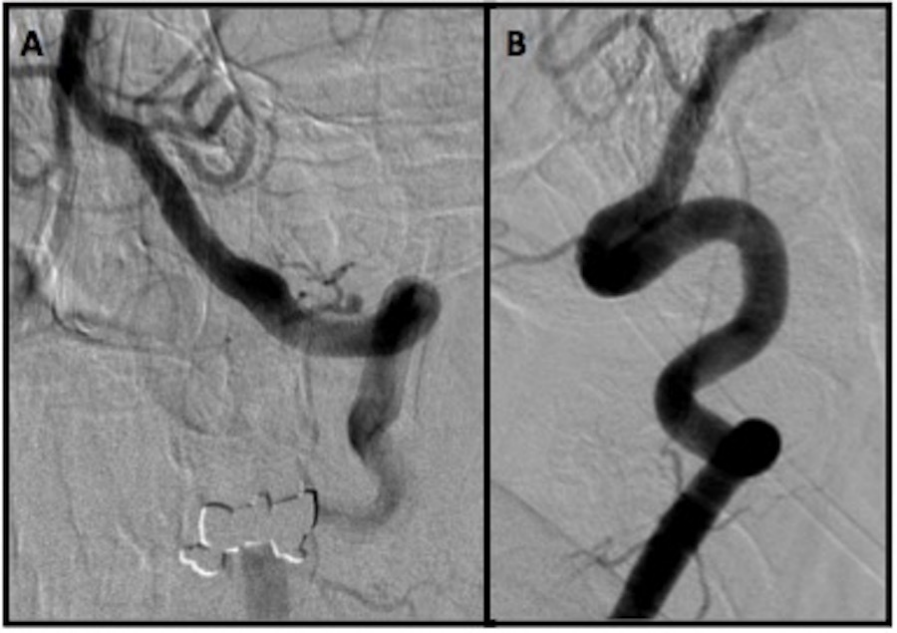
Vertebral Angiography Three Months After Treatment AP and lateral projections of the left vertebral angiography three months after treatment with pipeline embolization device. The pseudoaneurysm has been obliterated completely.

The patient was a 71-year-old male with a non-union C2 fracture and persistent pain admitted to an outside hospital for a posterior cervical fusion with C1-C2 screw fixation. However, that procedure was prematurely aborted because of perforation of the V3 segment of the VA by the surgical screw. The surgeon encountered the sudden onset of profound bleeding, which was estimated to be 3 liters. The surgical site was packed after attempting primary hemostasis. After the blood transfusion and stabilization, the patient was transferred to our hospital and underwent vertebral artery angiography the day after spinal surgery, which confirmed the development of an iatrogenic pseudoaneurysm in his dominant VA (Figure 1). The contralateral VA was a small and non-dominant one, and therefore, we had to save the injured VA by using a flow diversion technique. For this purpose, the patient was premedicated with standard loading doses of aspirin and Plavix. Subsequently, using standard endovascular techniques, a 3 mm x 16 mm PED was successfully deployed across the pseudoaneurysm. Final angiography showed an immediate significant stagnation of the blood flow inside the pseudoaneurysm’s cavity, but not complete occlusion. After deployment of PED, the patient was observed for two days and then was transferred back to the original spine center and underwent cervical exploration and surgical gauze removal. No intraoperative bleeding was noted. The patient was regularly followed up; finally, after three months, a check angiography was performed, which showed a complete obliteration of the pseudoaneurysm (Figure 2).

### Literature review

Only two cases of iatrogenic pseudoaneurysms of the vertebral artery treated with the Pipeline Embolization Device have been reported in the English literature (Table 1). Both patients were asymptomatic at follow-up, and follow-up angiography demonstrated complete pseudoaneurysm obliteration with patent vertebral arteries.

**Table 1:** Reported Cases of Iatrogenic Pseudoaneurysms of the Vertebral Artery Treated by Pipeline Embolization Device in English Literature Abbreviations: CT: Computed Tomography, M: male, PED: Pipeline Embolization Device, VA: Vertebral Artery, DSA: Digital Subtraction Angiography

Authors and Year	Age (years), Sex	Presentation	Diagnosis	Study	Treatment	Outcome	Follow-up Angiography
Ambekar, et al., 2014	47, M	Pulsatile swelling at operative site postop day two after C1-C2 fixation using screws and rods	right VA pseudoaneurysm (V3 segment)	CT Angiogram	PED	Asymptomatic at 10 months follow-up	CT angiogram: complete pseudoaneurysm occlusion, patent right VA
Dolati, et al., 2015	71, M	Bleeding during C1-C2 screw fixation	left VA pseudoaneurysm (V3 segment)	DSA	PED	Asymptomatic at 3 months follow-up	DSA: complete pseudoaneurysm occlusion, patent left VA

## Discussion

Pseudoaneurysms are usually encased only by a friable layer of connective tissue rather than a true wall. Iatrogenic pseudoaneurysms can be either fusiform, which result from arterial dissection resulting in a thinned tunica adventitia layer and resultant vessel dilation, or saccular, which result from a focal transmural arterial wall lesion [15]. Once identified, pseudoaneurysms require treatment to prevent expansion and rupture.

The third segment of the vertebral artery (V3) is particularly vulnerable to iatrogenic injury due to its unprotected course adjacent to numerous bony structures. V3 exits the protective transverse process of C2, and courses laterally to enter the transverse foramen of C1 [16]. Next, it courses posteriorly around the lateral mass of C1 and inferior to the posterior atlanto-occipital membrane lateral to the cervicomedullary junction [16]. Finally, it courses superomedially to enter the dura and arachnoid and then becomes V4 (the final vertebral artery segment).

Anatomical variation of the vertebrobasilar system is very common, which may contribute to the associated morbidity of the VA pseudoaneurysm development and treatment [17]. Asymmetry due to a unilateral hypoplastic VA, extracranial VA occlusion, or VA ending in the posterior inferior cerebellar artery (PICA) may result in increased reliance on the contralateral VA. Therefore, extreme caution must be taken to identify anatomic variations in the vertebrobasilar system and disrupting the dominant VA during pseudoaneurysm repair. The anatomic variant, known as duplicated VA, also poses an increased risk for injury as they have multiple points of attachment, which can result in traction injury [18]. Due to anatomical variants of the VA course, the safe placement of transarticular screws (TAS) on at least one side is not possible in between 10% and 20% of patients [19-20]. For these patients, alternative methods of fixation and fusion, such as C1 lateral mass and C2 pedicle or pars screws, extension down to C3, halo vest immobilization with wiring and bone grafting, or unilateral TAS fixation should be attempted [21].

**Figure 3:**
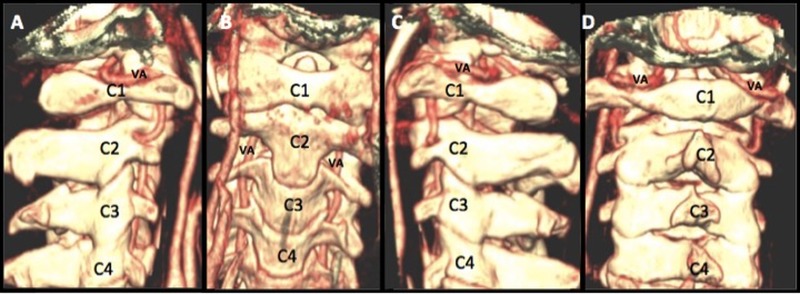
Tri-dimensional and Multiplanar Reconstructions of the Occiputo-Cervical Region Including VA and C1-C4 Vertebral Bodies Tri-dimensional and multiplanar reconstructions of the occiputo-cervical region, including VA and C1-C4 vertebral bodies in lateral, AP, and posterior projections (Panels A-D). By importing the computed tomography angiography images into any system with the tri-dimensional reconstruction capability, we will be able to visualize the occiputo-cervical bony structures with the VA in place. This gives us a 3D image of the position of the vertebral artery before starting screw fixation.

**Figure 4:**
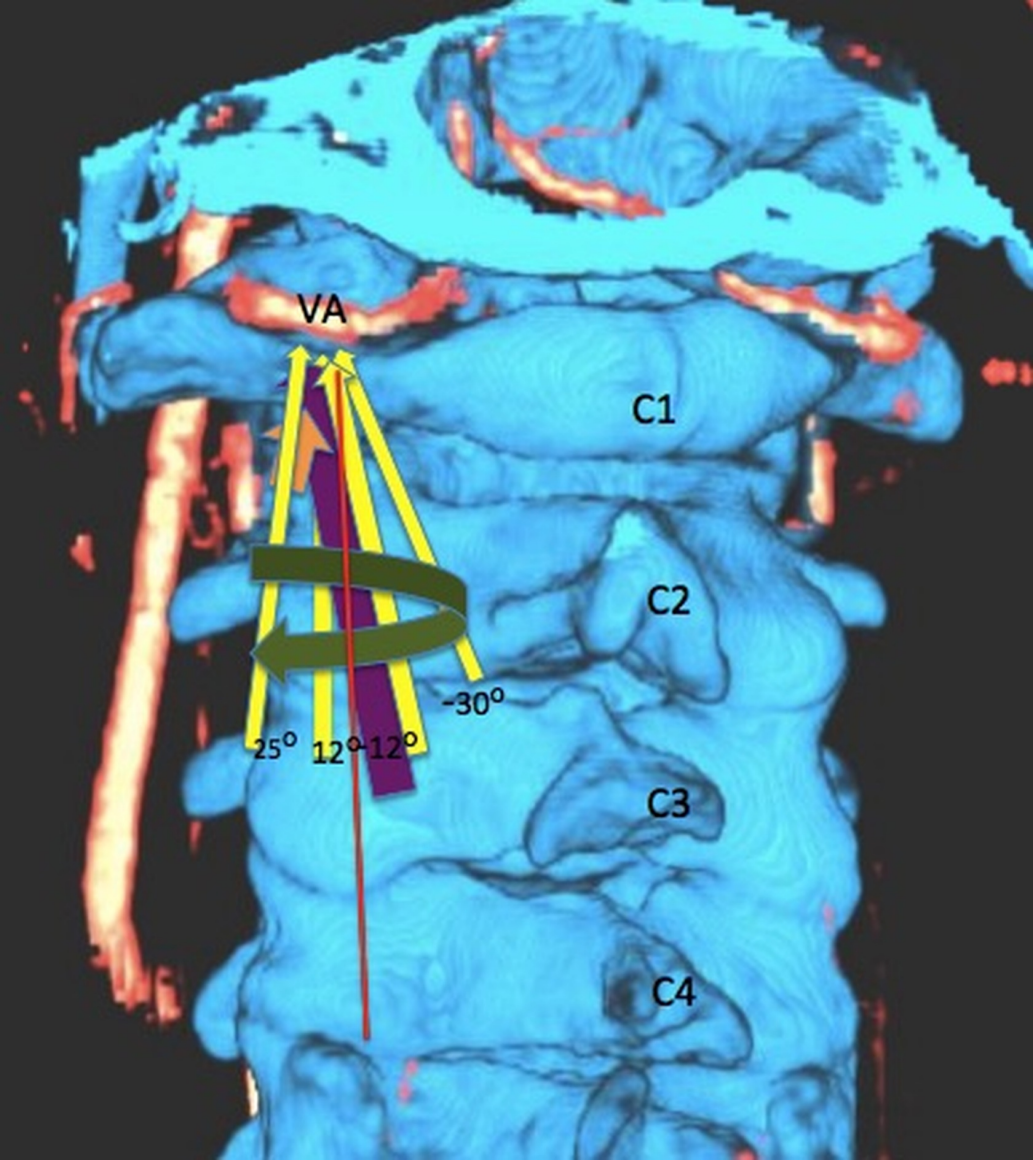
Three-dimensional Reconstruction of the Computed Tomography Angiography with Testing Different C1-C2 Fusion Screw Angle in Relation to the Vertebral Artery Three-dimensional reconstruction of the computed tomography angiography on the Siemens Leonardo system with testing different C1-C2 fusion screw angles in relation to the vertebral artery. This preoperative assessment of the entrance points and the screw’s angle penetration will help to realize anatomical variations and prevent VA injury.

The technique used for atlantoaxial fixation may also facilitate the development of a VA pseudoaneurysm. For example, one recent study showed that the most common cause of complications associated with this surgery were due to screw malposition [21]. This is concerning, as screw malposition occurred in 7% of patients [21], and VA injury has been reported to occur in between 0% and 8% of patients [2-7]. One study found a 1.7% incidence of VA injury per placed screw [4]. In addition to VA injury, screw malposition may lead to pseudarthrosis and damaged screws. The greatest risk factors for screw malposition include inadequate reduction of C1 on C2 leading to misalignment, lack of surgical experience, and insufficient knowledge of the patient’s anatomy [5, 7]. For these reasons, three-dimensional (3D) and multiplanar reconstructions of the local anatomy, including VA, C1, and C2, may be indicated for preoperative planning, determination of anatomic suitability [19], and potentially for intraoperative guidance of TAS placement (Figures 3-4). This tri-dimensional reconstruction from importing the images and performing the task usually takes 10-15 minutes.

Surgical treatment with ligation of pseudoaneurysms is considered the definitive treatment. However, it also is the most invasive of the procedures, so alternative strategies, such as endovascular treatments, are often sought to preserve the parent artery.

The most recently developed endovascular technique for aneurysm treatment is the micropore flow-diverting stent, which induces aneurysm thrombosis and resolution through alteration of the aneurysm hemodynamics. The two flow-diverting stents commercially available are the PED and the SILK (SFD, Balt Extrusion, Montmorency, France) [22]. Recently, PED has been shown to have a six-month aneurysm obliteration rate of 82.9% to 87.5%, with a periprocedural complication rate of 6.3% and 1.5% mortality rate [23-24]. While the SILK has been reported to achieve a similar aneurysm obliteration rate as the PED (approximately 80%), the SILK appears to be associated with a greater complication rate (approximately 17%) [25-26]. Insufficient studies on SILK have been reported to enable a direct comparison with PED. The PED has been shown to achieve flow diversion while maintaining the integrity of perforating arteries and parent artery branch vessels [19]. This property of the PED may be beneficial in treating VA pseudoaneurysms when we need to save important perforator arteries or main branches, such as the posterior inferior cerebellar or anterior spinal arteries.

While other endovascular techniques are available for aneurysm treatment, they possess disadvantages that flow-diverting stents were designed to overcome. For example, when pseudoaneurysms are treated with coil embolization, as there is no real wall for these aneurysms, the chance for rebleeding and intraoperative rupture and severe bleeding will be very high, especially in the acute setting of VA perforation by a screw. In contrast, the PED does not require intra-aneurysmal manipulation, and adjuvant coiling is not necessary. The PED also provides other theoretical advantages over other endovascular modalities. The PED enables full coverage of the aneurysm neck and implant, as it serves as scaffolding for growth of endothelial tissue [23]. Additionally, the PED has greater metal surface area coverage compared to balloon-expendable or self-expanding stents, which promotes neointimal regrowth and aneurysm neck occlusion [23].

Our experience mirrors that of Ambekar, et al. (2014), in that the PED can be used to successfully treat iatrogenic VA pseudoaneurysms [1]. We believe the PED is able to preserve parent vessel patency while excluding the pseudoaneurysm. Because flow-diversion stents require the absence of a large pressure gradient across the pseudoaneurysm wall to induce hemodynamic alteration and thrombosis, the PED is more likely to be effective if the pseudoaneurysm is not actively bleeding during treatment. Thus, the PED may not be successful and, in fact, may be contraindicated in treating acutely bleeding pseudoaneurysms.

Regardless, as flow diversion induces thrombosis over the time, rebleeding remains a potential complication in the short-term post-procedural period. Moreover, as a mandatory periprocedural requirement for the application of any intra-arterial stent, these patients need to be on a dual anti-platelets regimen to prevent intra-stent clot formation. This may interfere or delay the healing process and even may increase the chance of early postoperative bleeding. These may be the main limitations of the PED. Therefore, careful patient selection, close postoperative observation, and follow-up images are highly recommended.

## Conclusions

Iatrogenic pseudoaneurysms of the vertebral artery arising during cervical fusion, particularly in its V3 segment, are potential vascular lesions that are difficult to treat definitively. To our knowledge, this is the second case report of a successful application of a flow diversion stent for treatment of these pseudoaneurysms. PED is a useful tool in the management of such vascular lesions, leading to pseudoaneurysm isolation from the circulation without compromising parent vessel blood flow. However, pseudoaneurysm rupture remains a potential complication in the early post-procedural period. Therefore, close postoperative patient observation and interval follow-up images are highly recommended.
